# Walking together: behavioural signatures of psychological crowds

**DOI:** 10.1098/rsos.180172

**Published:** 2018-07-25

**Authors:** Anne Templeton, John Drury, Andrew Philippides

**Affiliations:** 1 School of Psychology, University of Kent, Canterbury, Kent CT2 7NP, UK; 2 School of Psychology, University of Sussex, Brighton, East Sussex, UK; 3 Department of Informatics, University of Sussex, Brighton, East Sussex, UK

**Keywords:** crowd movement, social identity, pedestrian movement, pedestrian modelling

## Abstract

Research in crowd psychology has demonstrated key differences between the behaviour of *physical* crowds where members are in the same place at the same time, and the collective behaviour of *psychological* crowds where the entire crowd perceive themselves to be part of the same group through a shared social identity. As yet, no research has investigated the behavioural effects that a shared social identity has on crowd movement at a pedestrian level. To investigate the direction and extent to which social identity influences the movement of crowds, 280 trajectories were tracked as participants walked in one of two conditions: (1) a psychological crowd primed to share a social identity; (2) a naturally occurring physical crowd. Behaviour was compared both within and between the conditions. In comparison to the physical crowd, members of the psychological crowd (i) walked slower, (ii) walked further, and (iii) maintained closer proximity. In addition, pedestrians who had to manoeuvre around the psychological crowd behaved differently to pedestrians who had to manoeuvre past the naturally occurring crowd. We conclude that the behavioural differences between physical and psychological crowds must be taken into account when considering crowd behaviour in event safety management and computer models of crowds.

## 1. Introduction

Coordinated crowd movement can be seen in numerous situations: a crowd of football fans celebrating together [1], pilgrims undertaking the Hajj in Saudi Arabia [2] and people in disasters coming together to support one another [3]. The complexity of crowd movement has made the underlying causes of crowd behaviour a source of fascination across multiple research disciplines. Crowd psychologists have attempted to look at the relationship between individuals and groups in influencing the perceptions and behaviour of the crowd (e.g. [4]). Computer modellers have researched the factors influencing pedestrian movement in order to create models which accurately predict movement in a variety of crowd scenarios, from evacuations [5,6], to pedestrian flow in crowded spaces [7–9]. Biologists have shown that we can gain insight to human crowd movement by looking to the behavioural patterns of social insects, fish and other non-human animals [10,11]. Additionally, physicists have demonstrated that crowd movement can be understood by comparing behaviour to particle physics and Newtonian forces [12,13]. While these disciplines may use separate paths to understand crowd movement, they share the goal of understanding crowd behaviour by exploring how people in crowds self-organize. Crowd psychology has shown that there are differences between *physical* crowds of co-present members, and the collective behaviour of *psychological* crowds where members act as a group due to their shared social identity. No research, however, has examined the behavioural effects social identities can have at a pedestrian movement level. This paper reports a study in which we examine the movement of crowds in one of two conditions: (1) a *psychological* crowd where the entire crowd is primed to share a social identity; (2) a naturally occurring *physical* crowd composed of small groups and individuals; and determine the factors underlying self-organizing behaviour in crowd movements by drawing on theories from social psychology.

### 1.1. Self-organization in crowds

The way in which crowds self-organize has been researched in four broad areas. First, the effect of socially transferred information on crowd movement has been examined in diverse disciplines. For example, research on birds, marine insects and fish has suggested that collective movement is influenced by non-verbal cues of velocity and the direction of movement of others [14], and knowledge of group structures based on cues from individuals [15]. Visual perception in human crowds has also been suggested to affect movements based on cues on where others in the crowd look [16,17] and walk [18]. A second focus has been the role of leadership and how crowds reach consensus decisions. For example, researchers have investigated how information is disseminated and how effectively crowds reach a target depending on which members of the crowd were informed [19–24]. Third, the influences of both macroscopic and microscopic level features of crowd behaviour on coordinated movement of the crowd have been analysed. Macroscopic computer models have examined the influence of factors such as density on pedestrian movement in emergency situations [25–27]. Conversely, microscopic modelling has examined the effect of an individual's movements on physical crowds, such as a pedestrian's motivation to avoid collisions [23,28] and their stepping behaviour [29,30].

An important growing fourth area of research is examining the effect of group behaviour on crowd movement. For instance, Moussaïd *et al*. [13] looked at the formations of approximately 1500 pedestrian groups in natural conditions to analyse their walking patterns and how groups influenced crowd flow, finding that small groups form ‘V’ formations as they move through the crowd. Research by Vizzari *et al.* [31] explored the role of groups on crowd flow by manipulating the size of group to be either a single pedestrian, three pairs of pedestrians, two groups of three pedestrians or two groups of six pedestrians. This unique experiment told the pedestrians in the group conditions to stay together as friends or relatives would, and found that when the groups tried to maintain a formation it increased their travel time. The effects of groups in crowds have also been applied to affiliation behaviour in evacuations [32], egress [33–35] and the walking formations of groups in crowds [36,37].

Crucially, however, these studies investigate subgroups within a crowd rather than when an entire crowd acts as a group, nor, with the exception of Vizzari *et al*. [31], do they analyse what makes a ‘group’. Indeed, very few studies on the self-organization of crowds have examined the psychological underpinnings of what a ‘crowd’ is and how this could influence movement. Such an understanding is needed to explain why one type of crowd exhibits greater, or different, self-organizing collective behaviour compared to another. One social psychological approach that has shown that there are key differences between crowds that share a social identity and those that do not, and can elucidate whether and how social psychological factors may influence crowd self-organization, is self-categorization theory (SCT) [38].

### 1.2. Defining the ‘crowd’

Understanding the psychology of a crowd can help explain important behavioural differences between, for example, a crowd of commuters walking during rush hour and a crowd of sightseeing tourists who coordinate their behaviour to remain together. Reicher [39] distinguishes between *physical* crowds, which are composed of individuals who are physically co-present but do not share a sense of being in the same group (such as the commuters), and *psychological* crowds where members also share a sense of ‘group-ness’ (such as the sightseeing tourists who see themselves as a group). SCT can explain this distinction and demonstrates that physical aggregates of individuals can become a psychological group through the process of depersonalization: individuals self-stereotype themselves as being in a group, so that they shift from their personal identity to identifying as a member of a group [38]. It is through this *shared social identity* that collective behaviour becomes possible [40].

SCT has been applied to a multitude of crowd scenarios to show how social identity can explain features of psychological crowds, such as feelings of safety during the Hajj [2], people coordinating their actions in an emergency evacuation [3,41–43], and intimacy behaviours [44]. However, only a limited number of studies have examined predictions for the behavioural consequences of shared social identity in a crowd, and none have applied the principles to modelling pedestrian behaviour. Indeed, one of the key behavioural predictions of SCT—that ingroup members will remain together based on their shared social identities—is yet to be quantified in large crowd behaviour.

Experimental research has examined the extent to which social identity can affect behaviour such as the maintenance of physical distance (or proximity) between small groups of people. Research by Novelli *et al.* [45] found that when participants defined themselves as being in the same group as another person in the room, the participants moved their chairs significantly closer together than if the other person was perceived to be a member of a different group. Crucially, Drury *et al.* [41] found that survivors of the 2005 London bombings became a psychological crowd in the aftermath of the bombs and remained together to help one another. We suggest that these findings can be used to derive predictions about the effect of social identity on proximity behaviours in walking crowds: specifically, those who are in the same group are willing to be closer to one another and will therefore try to stay together, which will have consequences for flow rates.

Given the findings from social psychology that people with a shared social identity coordinate their behaviour and are willing to be physically closer to ingroup members, our research investigates the effect of social identity on the movement of psychological crowds compared to physical crowds. We argue that due to ingroup members attempting to remain together, there are distinct behavioural signatures which distinguish psychological crowds from physical crowds, and that these are explicable in terms of shared social identity. Using minimal group manipulation techniques from social psychology [46], we compare the walking behaviour of a psychological crowd and a physical crowd to assess the effect a shared social identity has on walking behaviour. In particular, we analyse differences in walking speed, distance, and proximity between the crowds. We hypothesize that shared social identity will cause members of the psychological crowd to (1) alter their speed to remain with other psychological crowd members, (2) alter the distance walked to remain together, and (3) stay together by (3a) maintaining closer proximity and (3b) walk in larger subgroups than in the physical crowds.

## 2. Methodology

### 2.1. Design and materials

A field study of walking behaviour in two crowds was conducted at the University of Sussex campus in England. In the experimental condition, a *psychological* crowd was created by priming participants to share a social identity. These participants (*N* = 120) signed up to be part of a study on walking behaviour and were selected based on their attendance of a second year Psychology class. A shared social identity amongst participants was evoked using standard forms of social identity manipulation [46]: we provided every participant in the psychological crowd with an identity prime of a black baseball cap with a ‘Sussex Psychology’ logo on it. This logo was emblematic of a social identity already available to each participant and was used to make that social identity salient. It also enabled participants to see who else was in their group and allowed the experimenters to track who had been primed to share a social identity. Each participant was asked to walk from the lecture to a nearby location on campus. Around these recruited participants were an additional 47 pedestrians walking in the same area.

One week prior to the experimental condition, we filmed 121 people, who were primarily composed of the same second year Psychology students at Sussex as they left their lecture to walk to the other side of campus. This was a naturally occurring *physical* crowd, as the participants were not manipulated. We ensured that the person filming was visible by wearing a high visibility jacket and filming from a low bridge directly above the path the crowd walked under. We attempted to keep the conditions as similar as possible within the limits of fieldwork. Both crowds were filmed at the same time of day a fortnight apart in the same weather conditions (sunny) after their lecture to ensure they had the same timetable commitments. Importantly, participants in both the psychological and physical crowd conditions largely comprised the same people to ensure that any pre-existing relationships between the crowd members were the same before priming the crowd to share a social identity, thus keeping any friendship groups consistent in both conditions.

Filming was performed with a Nikon PixPro AZ361 digital camera with a 36× wide 24–864 mm equivalent Aspheric HD Zoom Lens with no zoom or lens distortion. We filmed the participants from above to aid participant tracking as they walked along a section of the path on the route (we filmed an area 10 m in length and 3.75 m in width), with the camera set up at the centre of a low bridge crossing the path perpendicularly. We selected this path as it is an area where students walk between lectures and the main campus, and, by keeping conditions as similar as possible, hoped that the participants would be met with similar counterflow pedestrians around both crowds. There were 55 people in counterflow to the physical crowd, and 34 people in counterflow to the psychological crowd. Additionally, there were 13 people walking the same direction as the psychological crowd in that condition, but on the other side of the path to those walking in counterflow.

**Figure 1.**
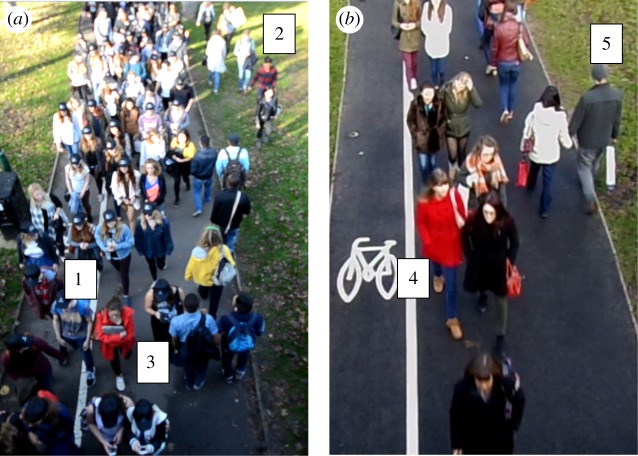
Snapshots of the pedestrians in both crowd conditions. Picture (*a*) depicts the experimental condition where Group 1 are the recruited participants, Group 2 are the pedestrians walking in the same direction and Group 3 are the pedestrians walking in counterflow. Picture (*b*) depicts the control condition with naturally occurring crowd behaviour, where Group 4 are those walking towards the camera and Group 5 are those walking away from the camera.

To enable between-groups analysis, those in the footage were classified as follows: participants primed to share a social identity were classified as Group 1 (*N* = 112); the people who were not recruited and were walking in the same direction as the psychological crowd (towards the camera) were classified as Group 2 (*N* = 13), and those who were walking in counterflow to the crowd (away from the camera) were classified as Group 3 (*N* = 34). Within the control condition, those walking towards the camera were classified as Group 4 (*N* = 66), and those walking away from the camera were classified as Group 5 (*N* = 55) (figure 1).

### 2.2. Trajectory analysis

The positions of the crowd members were extracted using custom-made MATLAB software which allowed manual selection of each participant every five frames (frame rate 24 frames per second), to reconstruct their trajectories as they walked throughout the footage. Head positions were tracked because the pedestrians' positions on the ground could not be derived from the pedestrians' feet positions, as these were not always visible due to the density of the crowd and angle of filming. The data were transformed from the camera angle above the bridge to a directly top-down planar view in order to assess the locations of the pedestrians on the ground, defined to be approximately the centre-of-mass of their bodies. The transformation matrix was derived by selecting corners of a 3.75 m by 5 m rectangle painted on the ground, and the proceeding transformation matrix provided the real-world coordinates of the path the participants walked along.

**Figure 2.**
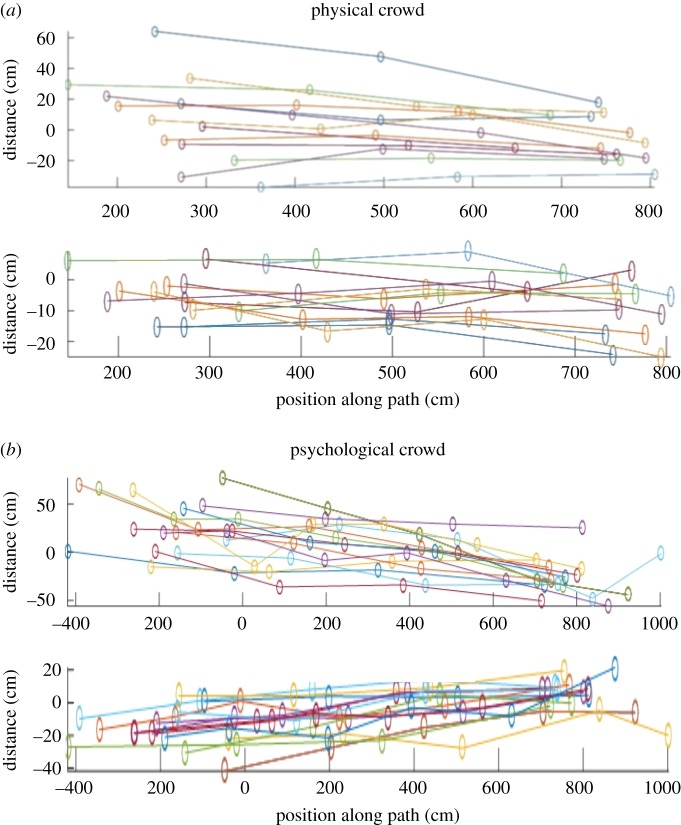
The distance between projected feet positions and actual feet positions of 14 pedestrians in each crowd. The *X* axis denotes the movement of the pedestrians from the beginning of the path to the end, with each circle indicating their tracked positions. The *Y* axis denotes the distance between the projected positions of the feet and the actual positions of the feet from manual tracking. The top figures in each crowd demonstrate the error of projections perpendicular to the camera (error to the left or right side of the pedestrians). The bottom figures demonstrate the error of projections parallel to the camera (in front or behind the pedestrians).

To perform the transformation to a planar view, we assumed a constant height for the participants of 169 cm (which is the average of the average heights of UK men and women) and that their heads were directly above their centre-of-mass. This process will lead to errors from swaying of heads and height differences. To quantify the extent of these errors, we used a sample of participants whose feet were visible, and compared the planar positions derived from their feet positions (the average position of their feet) to the planar positions derived from head positions. Differences are shown in figure 2. While there are some large differences, the median and interquartile ranges for the differences are 18 ± 13 cm for the physical crowd, and 28 ± 17 cm for the psychological crowd. Importantly, the differences within participants' trajectories are consistent, suggesting that the differences are predominantly caused by height variation between participants. This is reinforced by the fact that errors are greater in the *y*-axis which is perpendicular to the camera plane and decrease as the participants come towards the camera. Since the errors are approximately consistent within each trajectory, they do not affect measures of speed and distance travelled.

The pedestrians' projected feet positions were then used to ascertain their walking speed, distance walked, and the proximity between individuals. Speed for each pedestrian was calculated as distance/time, where time = 0.2085 = 1 second divided by frame rate multiplied by 5 (as 5 is the frame gap used when tracking trajectories). The distance each pedestrian walked was calculated by summing the distance between the coordinates of each step, as the tracked positions of the transformation matrix represented the actual distance the participants walked. The space around each pedestrian was measured using Voronoi tessellation areas which sets a polygon around each member of the crowd based on the distance to their nearest neighbours at each time point. These areas were calculated using Sievers's [47] method for Voronoi decomposition and implemented in MATLAB, with vertices constrained so that the maximum tessellation area radius is 1 m to avoid artificially inflating the space around individuals walking alone or on the periphery of the crowd.

To ascertain how much space individuals maintained around them, the footage of both crowds was sliced into time-points to get snapshots of the pedestrian locations every 4.17 s (100 frames), producing 10 time-points for each condition and spanning the entirety of the psychological crowd footage. One possible issue is that there were different numbers of people at different time-points in the experimental condition compared to the control condition, and the number of people around the psychological crowd changes as they walk through the footage. As such, latent growth curve analysis was used in R to determine (1) whether there were differences in tessellation areas between groups, (2) whether their tessellation areas changed over time, and (3) whether this was affected by the number of people in the area.

Following this, a prima facie analysis was conducted to determine how pedestrian groups maintained formation while walking. Hierarchical agglomerative cluster analysis was used with between-groups linkage, Euclidian distance and standardized *z*-scores, to group participants based on the distance between their locations at the different time-points. This explored whether the crowds split into smaller groups through classifying sub-groups (or clusters) by examining the optimum number of clusters within each time-point. We then also compared which participants were in clusters in successive time-points to ascertain whether clusters remained together.

## 3. Results

### 3.1. Speed of movement

**Figure 3.**
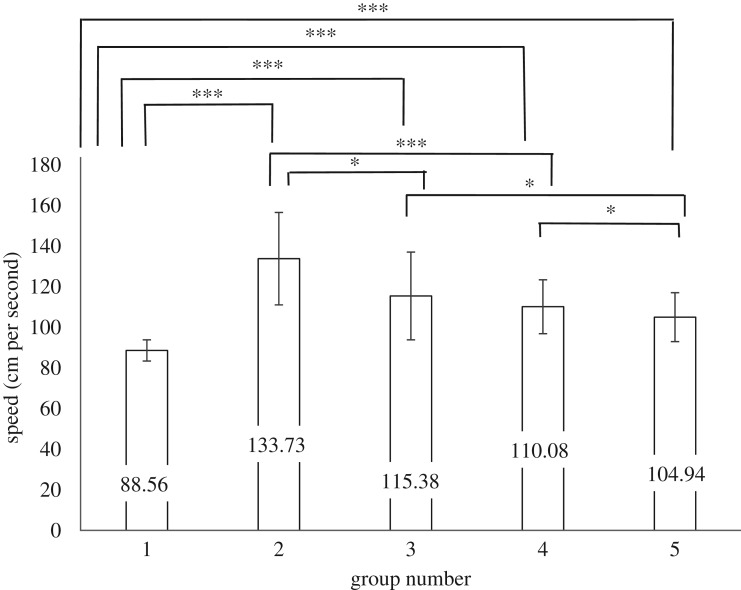
Means and standard deviations for the speed each group walked. Significant differences from the between-groups comparison are depicted by corresponding overhead lines. Group 1 s.e. = 0.491; Group 2 s.e. = 6.304; Group 3 s.e. = 3.702, Group 4 s.e. = 1.633; Group 5 s.e. = 1.625. * indicates *p* < 0.05; *** indicates *p* < 0.001.

Kolmogorov–Smirnov tests revealed that Groups 1, 2, 3 and 5 did not significantly deviate from normal distribution, but Group 4 was non-normally distributed (see table 1 for *D* values, d.f. and *p*-values, and figure 3 for means and standard errors). Independent *t*-tests were used to compare groups which were parametric, and Kruskal–Wallis *H* tests were used to compare groups where one or both groups were non-parametric.

**Table 1.** Kolmogorov–Smirnov tests for each group for speed of movement, distance walked and tessellation areas. Non-normal distributions are indicated in bold.

	speed			distance			tessellation areas		
	*D*	d.f.	*p*	*D*	d.f.	*p*	*D*	d.f.	*p*
Group 1	0.060	112	0.200	0.096	112	**0**.**014**	0.104	418	**0**.**001**
Group 2	0.147	13	0.200	0.285	13	**0**.**005**	0.119	25	0.200
Group 3	0.111	34	0.200	0.337	34	**0**.**001**	0.066	56	0.200
Group 4	0.149	66	**0**.**001**	0.067	66	0.200	0.067	47	0.200
Group 5	0.083	55	0.200	0.079	55	0.200	0.102	52	0.200

When comparing the groups within conditions, on average, Group 1 walked significantly slower than those in Group 2 (walking in the same direction as Group 1), −45.170, BCa 95% CI [−58.929, −31.411], *t*_12.146_ = −7.134, *p *< 0.001, *r* = 0.899. Group 1 also walked significantly slower than those in Group 3 (in counterflow to Group 1), −26.824, BCa 95% CI [−34.411, −19.236], *t*_34.170_ = −7.183, *p *< 0.001, *r* = 0.776. On average, Group 2 walked quicker than Group 3, −18.345, BCa 95% CI [3.965, 32.728], *t*_45_ = 2.569, *p *= 0.014, *r* = 0.358. In the control condition, Group 4 (mean rank = 66.88) walked significantly faster than Group 5 (those walking in counterflow to Group 4, mean rank = 53.95), *H*_1_ = 5.186, *p *= 0.023.

When comparing the group across crowd conditions, crucially, on average participants walked significantly more slowly when they were primed to share social identity (Group 1, mean rank = 58.30) than when they were not (Group 4, mean rank = 142.44), *H*_1_ = 110.720, *p *< 0.001. An independent *t*-test found that Group 1 also moved significantly slower than those in counterflow in the control condition (Group 5), −16.389, BCa 95% CI [−19.778, 12.998], *t*_64.077_ = −9.658, *p *< 0.001. Those going around the psychological crowd (Group 2, mean rank = 59.54) walked faster than those going the same direction in the control crowd (Group 4, mean rank = 36.15), *H*_1_ = 11.279, *p *< 0.001, suggesting that the psychological crowd has an effect on people walking in the same area due to manoeuvring around it. This is also found when comparing those in counterflow to the psychological crowd (Group 3) who walked significantly faster than those walking the same direction in the control condition (Group 5), −10.436, BCa 95% CI [2.298, 18.573], *t*_45.888_ = 2.581, *p *= 0.013, *r* = .356. Overall, these results confirm Hypothesis 1.

### 3.2. Distance

**Figure 4.**
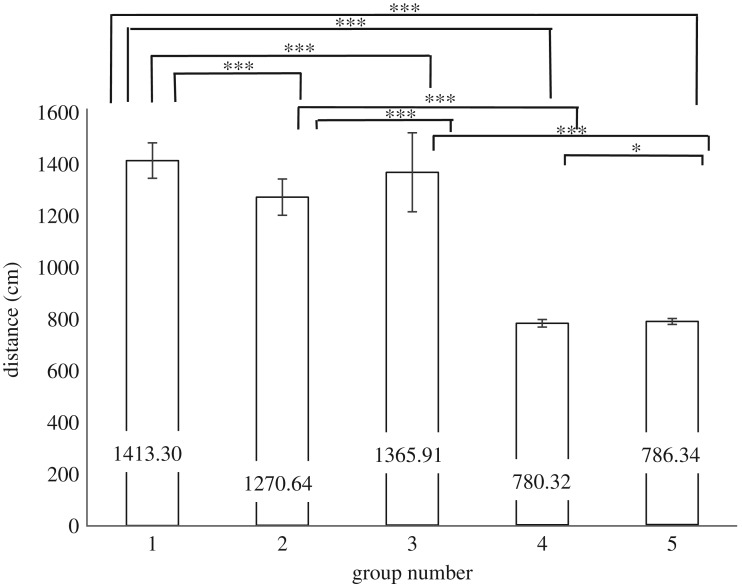
Means and standard errors for the distance each group walked in cm. Significant differences from the between-groups comparison tests are depicted by corresponding overhead lines. Group 1 s.e. = 6.472; Group 2 s.e. = 19.521; Group 3 s.e. = 26.195, Group 4 s.e. = 1.192; Group 5 s.e. = 1.561. * indicates *p* < 0.05; *** indicates *p* < 0.001.

Kolmogorov–Smirnov tests revealed the distance of Groups 1, 2 and 3 were non-normally distributed, but Groups 4 and 5 did not deviate significantly from normal (see table 1 for *D* values, d.f. and *p*-values, see figure 4 for means and standard errors). Again, independent *t*-tests were used to compare groups which were parametric, and Kruskal–Wallis *H* tests were used to compare groups where one or both groups were non-parametric.

Between-groups analysis for groups within conditions showed that participants in Group 1 (mean rank = 68.49) walked significantly further when compared to Group 2 (mean rank = 15.69), *H*_1_ = 24.734, *p *< 0.001, and when Group 1 (mean rank = 83.08) was compared to Group 3 (mean rank = 41.94), *H*_1_ = 24.683, *p *<  0.001*.* Group 3 (mean rank = 28.88) also walked significantly further than Group 2 (mean rank = 11.23), *H*_1_ = 15.586, *p *< 0.001, possibly due to Group 3 being in counterflow with Group 1 and 2 so having to manoeuvre around them. In the control condition, Group 4 walked significantly further than Group 5, −6.021, BCa 95% CI [−10.825, −1.218], *t*_119_ = −2.482, *p *= 0.014, *r* = 0.05.

Comparisons across crowd conditions found that Group 1 (mean rank = 122) walked significantly further than Group 4 (mean rank = 35.50), *H*_1_ = 123.476, *p *< 0.001*,* supporting Hypothesis 2 that those who share a social identity walked further in order to remain together. Group 1 (mean rank = 111.50) also walked faster than Group 5 (mean rank = 28), *H*_1_ = 110.005, *p *< 0.001. Group 2 (mean rank = 73) walked significantly further than Group 4 (mean rank = 33.50), *H*_1_ = 32.175, *p* < 0.001. Group 3 (mean rank = 72.50) also walked significantly further than its counterpart in the control condition, Group 5 (mean rank = 28), *H*_1_ = 62.333, *p *< 0.001, again suggesting that the psychological crowd affected those around it.

### 3.3. Proximity

#### 3.3.1. Distance measures

**Figure 5.**
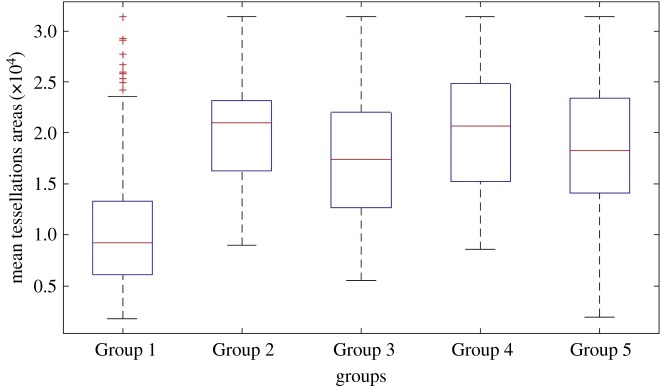
Box and whisker plots show the distribution of tessellation areas for the different groups gathered over 10 time-points. Red lines indicate the medians, boxes cover the 25th and 75th percentile and whiskers extend to 1.5 times the inter-quartile range. Red plus symbols indicate outliers. The mean tessellation areas for each group across all time points were, Group 1: *M* = 10383.29, s.d. = 5503.682; Group 2: *M* = 20218.67, s.d. = 5626.12; Group 3: *M* = 17732.70, s.d. = 6493.58; Group 4: *M* = 20506.39, s.d. = 6404.64, Group 5: *M* = 18298.48, s.d. = 7006.30.

Kolmogorov–Smirnov tests indicated the mean tessellation areas of Group 1 were non-normally distributed, but all others groups did not deviate significantly from normal (see table 1 for *D*-values, d.f. and *p*-values). The mean tessellation areas for each group across all time points were, Group 1: *M* = 10383.29, s.d. = 5503.68; Group 2: *M* = 20218.67, s.d. = 5626.12; Group 3: *M* = 17732.70, s.d. = 6493.58; Group 4: *M* = 20506.39, s.d. = 6404.64, Group 5: *M* = 18298.48, s.d. = 7006.30. Please see figure 5 for group medians and standard deviations, where red lines indicate the medians, boxes cover the 25th and 75th percentile and whiskers extend to 1.5 times the inter-quartile range, and red plus symbols indicate outliers.

Between-groups analysis was conducted on the mean tessellation areas across all time points. The tessellation areas of people in Group 1 were significantly smaller than those for people in all other groups, supporting our Hypothesis 3a. Group 1 (mean rank = 212.23) has significantly smaller tessellation areas than Group 2 (mean rank = 385.32), *H*_1_ = 43.11, *p* < 0.001; and Group 3 (Group 1 mean rank = 220.10, Group 3 mean rank = 367.38), *H*_1_ = 57.10, *p* < 0.001; and Group 4 (Group 1 mean rank = 214.95, Group 4 mean rank = 393.54), *H*_1_ = 74.63, *p* < 0.001; and Group 5 (Group 1 mean rank = 218.65, Group 5 mean rank = 370.94), *H*_1_ = 58.14, *p* < 0.001, showing that those in the psychological crowd maintained less space around them. A one-way ANOVA demonstrated that all other between-groups comparisons were non-significant suggesting there was no effect of group on tessellation size, *F*_3, 176_ = 2.13, *p *= 0.099, *w* = 0.135. The linear trend was non-significant, *F*_1, 176_ = 0.38, *p *= 0.536, *w* = 0.171, indicating no proportional change with group number.

Latent growth curve modelling was used to predict (1) the effect of group on tessellation areas, (2) the effect of group on changes in tessellation areas over three time-points, and (3) the effect of number of people on the tessellation areas. We used the tessellation areas of participants from when their first tessellation area was calculated (Time 1), and their tessellation areas at the following two time-points (Time 2 and Time 3). The intercept was weighted as 1 on each time-point to constrain them as equal. The slope was weighted on the time-points as Time 1_0_, Time 2_1_ and Time 3_2_ as the times were equally spaced at 4.17 s apart. The intercept and slopes were extracted across Time 1, Time 2, and Time 3 and used as estimates of (1) baseline tessellation areas and (2) increase or decline in tessellation areas across the successive time-points. We allowed a direct relationship between the number of people in the area at each time-point and the corresponding tessellation areas of the participants at those time-points. Group was regressed on to the intercept and slope, and participants were coded in their relevant groups. Robust maximum likelihood and full information maximum-likelihood (FIML) were used for missing data in Time 3 as the faster speed of pedestrians in Groups 4 and 5 meant that some participants could only be tracked across two time-points.

**Figure 6.**
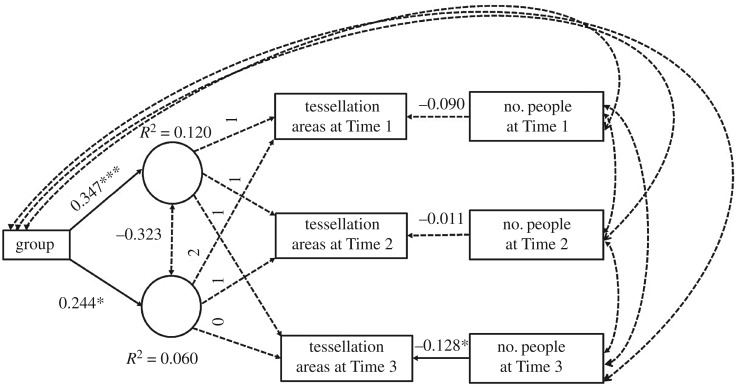
Results for latent growth curve modelling with standardized estimates indicating tessellation areas as a function of group and number of people in the area, and *R*^2^ values for the intercept, slope, Time 1, Time 2 and Time 3. Solid lines indicate significant pathways, and dotted lines indicate non-significant pathways (*p < 0.05, ***p < 0.001).

**Figure 7.**
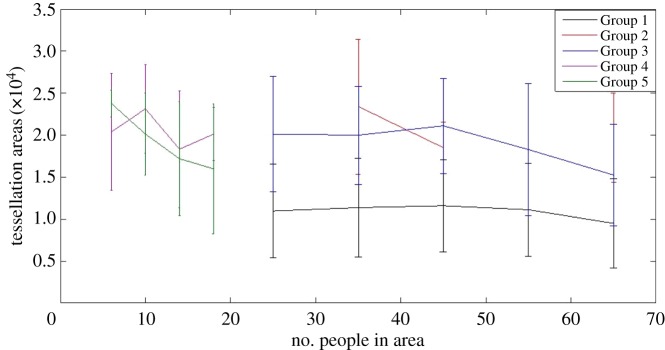
The median tessellation areas of each group as the number of pedestrians increases. Data are binned according to the number of people. For each bin, lines show the median values, while error bars indicate the 25th and 75th percentiles of the data.

We used the criteria suggested by Hu & Bentler [48] to assess model fit, which suggests RMSEA < 0.06, SRMR < 0.08, CFI > 0.95. This led us to consider our model provided adequate fit, RMSEA = 0.073, SRMR = 0.075, CFI = 0.982. Notably, chi-squared was non-significant, $\chi_{7}^{2}=11.604$, *p* = 0.114. In the model, the number of people was a non-significant predictor on tessellation areas at Time 1, *β* = 0.09, *p *= 0.167, and Time 2, *β* = 0.011, *p *= 0.167, but was a significant predictor at Time 3, *β* = 0.128, *p *= 0.024, which had the highest number of people. The groups have significantly different initial tessellation areas at Time 1, *β* = 0.35, *p* < 0.001, with people in Groups 2, 3, 4 and 5 appearing to have larger initial tessellation areas. Group was a significant predictor of change over time, *β* = 0.25, *p *= 0.029, indicating that the change of tessellation areas over time were different for the groups when including the number of people in the area (see figure 6 for path diagram and *R*^2^ values). As can be seen in figure 7, as the number of people increases the tessellation areas were affected in Groups 2, 3, 4 and 5, but the tessellation areas for Group 1 remained mostly constant regardless of the number of people in the area. This indicates support for our Hypothesis 3a that those who shared a social identity remained in closer proximity even when there was space available to spread out.

#### 3.3.2. Subgroup size

**Figure 8.**
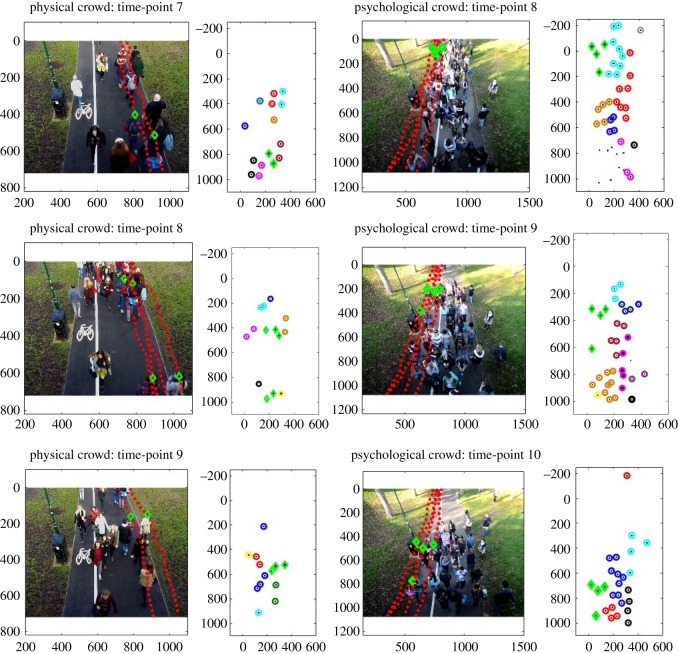
The progression of groups identified by cluster analysis over three time points in the physical and psychological crowds. Green diamonds denote pedestrians whose trajectories across the entire footage have been demonstrated. The progression of one group is shown in the psychological crowd, but two groups are shown in the physical crowd due to the faster walking pace of the pedestrians meaning that they could not be tracked across all three time-points (note that there are two groups shown in time-point 8 of the physical crowd).

Cluster analysis compared the number of subgroups within each group and found that those with a shared social identity (Group 1) walked in larger subgroups. The largest clusters in Groups 4 and 5 comprised three people, compared to clusters of 11 in Group 1. Moreover, the subgroups typically remained together while walking along the path throughout the progression of the time points, supporting our Hypothesis 3b that the psychological crowd would remain together in larger groups than in the physical crowd when they were not primed to share a social identity (figure 8). This provides prima facie support for our Hypothesis 3b that larger subgroups occur and are maintained in the psychological crowd, rather than splitting into the smaller groups that can be seen in physical crowds.

## 4. Discussion

By priming a crowd to share a social identity and comparing their behaviour to a naturally occurring crowd, we show core behavioural differences between psychological and physical crowds. We demonstrate that a shared sense of social identity motivated more coordinated behaviour amongst the participants. First, the psychological crowd walked slower than the other groups. Second, they walked further than the other groups. Third, they maintained closer proximity regardless of the number of people in the area. Fourth, they consisted of larger groups within the crowd and did not split into the small clusters seen in the physical crowd.

Further, those who had to manoeuvre around the psychological crowd walked faster and walked further than when no psychological crowd was present (even when in counterflow), while people walking the same direction as the psychological crowd maintained more distance around themselves than people in the physical crowd condition. This is additional but complementary to our hypotheses, and suggests that when a large psychological crowd was present, those outside it change their behaviour in order to avoid walking through the crowd.

These behavioural patterns have implications for understanding the self-organizing behaviour of psychological crowds. Research in social psychology has shown that numerous crowds with shared social identities exhibit self-organizing behaviour and would be considered a psychological crowd as defined in this study. For example, at the Hajj when pilgrims coordinate their behaviour to perform rituals in potentially dangerous densities [2], or when a physical crowd become a psychological crowd in an emergency and form orderly lines to evacuate, and let others go first and stay back to help people who are injured [3]. Here we provide quantified behavioural signatures of the movements of both psychological and physical crowds, showing how a shared social identity leads to different behaviours.

To our knowledge, there is no group-specific norm among Sussex Psychology students of walking in close proximity. As such, our findings can be extrapolated to other psychological crowds and have particular relevance to research on the effect of information transference and leadership in crowd behaviour, as we demonstrate that social identity has an effect on self-organizing behaviour in psychological crowds. In contrast to previous literature (such as [19,21,23]), in our study we provided no leader or information other than the location they were directed to, which group the members were in, and who else was in their group (indicated by the identity markers on their hats). Having identity markers as a source of information for crowd members might be thought to be artificial, but it is seen in other crowd events, such as sporting events where fans wear team memorabilia, or music events where attendees wear band emblems. The shared social identity manipulation was the principal difference between the two conditions and thus, we argue, the main cause of the coordinated behaviour. While research on leadership and transference of information may be applied to physical crowds, our results suggest that leadership is not necessary for self-organized coordination in psychological crowds.

People walking in counterflow to the psychological crowd, rather than attempting to walk through the psychological crowd, steered to the side of the crowd and walked in counterflow between the psychological crowd and those who were walking in the same direction as the psychological crowd. This could indicate that they treated the psychological crowd as one group and could distinguish between the psychological crowd and those in Group 2 who were walking in the same direction. Similarly, rather than joining the psychological crowd, Group 2 avoided the crowd and moved around it, indicating that they too perceived the crowd as an entity due to the coordinated behaviour. We thus suggest that a psychological crowd may cause people around the crowd to walk differently than when a merely physical crowd is present. However, one limitation of the present study is that these avoidance behaviours could be due to the lack of available room to walk in (due to the higher density of Group 1 than all other groups) rather than perception of the psychological group as a whole. Future research should examine whether the psychological crowd was perceived as an entity by outsiders, and whether the same behaviour occurs when there is more space available for the pedestrians to avoid the crowd.

In previous research, social identity has been shown to affect how a crowd interacts in emergency evacuations, such as survivors stopping to stay with and help others in their group, therefore delaying evacuation time [3,37]. Our results indicate that the crowd members may cluster together even when there is space available. The decreased walking speed of the psychological crowd supports the findings of Vizzari *et al*. [31] that the speed of groups is reduced when they attempt to keep formation. This is an important consideration for safety planning of crowd events and crowd models that assume crowds will split up into smaller subgroups [33]. Our results suggest that when a shared social identity is salient, the members of the crowd may remain in larger groups rather than splitting up or acting as individuals, as we observed in the physical crowd. Future research could extend this principle to crowd safety to explore the effect of social identity on cluster sizes within crowds, and how large clusters remaining together affects ingress and egress time.

As Reuter *et al*. [37] indicate, computer models are increasingly being used to plan for crowd behaviour in public spaces, and to do this safely they must be validated using real-world data. However, a recent systematic review of crowd simulations [49] found that, as yet, modellers have not incorporated the different behaviour of psychological crowds where an entire crowd shares a social identity. Here we quantify how social identity influences the behaviour of people in psychological crowds, indicating that it should be considered in interpretations of self-organizing crowd behaviour. The differences in speed, distance and proximity are crucial factors to consider when planning how a crowd will behave during ingress, egress, or in the event of an emergency situation. Crowd safety professionals and crowd modellers should thus develop crowd planning and simulations that distinguish the behavioural signatures of psychological and physical crowds in order to accurately replicate these different behavioural patterns.

## Supplementary Material

Tesselation_areas

## Supplementary Material

Speed_and_distance

## Supplementary Material

Cluster_Data

## Data accessibility

Raw data to replicate the analysis for speed and distance is included as electronic supplementary material in the SPSS file ‘Speed_and_distance’. This contains the speed, distance and group number for each participant. The raw data for the cluster analysis is included as electronic supplementary material in the SPSS file ‘Cluster_Data’ which includes the x and y coordinates for each participant and the corresponding time-points and group numbers. The raw data for the latent growth curve modelling is included as electronic supplementary material in the SPSS file ‘Tessellation_Areas’, which includes the following information for each participant: the tessellation area, group number time-point, and number of people present in the footage at the corresponding time-point to be used as a covariate. For access to the footage, interested parties should contact the corresponding author. This has not been uploaded to maintain the anonymity of the participants. Please note that if the footage is requested then the original footage will need to be slightly blurred to avoid facial identification of the participants.

## Licence to publish and open access

The authors grant a Licence to Publish under the CC-BY licence.
